# Liver Stiffness Measurement in Psoriasis: Do Metabolic or Disease Factors Play the Important Role?

**DOI:** 10.1155/2016/7963972

**Published:** 2016-02-23

**Authors:** Jamrus Pongpit, Saneerat Porntharukchareon, Piyaporn Kaewduang, Kwannapa Promson, Wasana Stitchantrakul, Supanna Petraksa, Ammarin Thakkinstian, Chomsri Kositchaiwat, Natta Rajatanavin, Abhasnee Sobhonslidsuk

**Affiliations:** ^1^Division of Gastroenterology and Hepatology, Department of Medicine, Faculty of Medicine, Ramathibodi Hospital, Mahidol University, Bangkok 10400, Thailand; ^2^Division of Dermatology, Department of Medicine, Faculty of Medicine, Ramathibodi Hospital, Mahidol University, Bangkok 10400, Thailand; ^3^Research Center, Faculty of Medicine, Ramathibodi Hospital, Mahidol University, Bangkok 10400, Thailand; ^4^Section for Clinical Epidemiology and Biostatistics, Faculty of Medicine, Ramathibodi Hospital, Mahidol University, Bangkok 10400, Thailand

## Abstract

*Background*. An increased prevalence of metabolic syndrome including nonalcoholic fatty liver disease (NAFLD) was reported in psoriasis. NAFLD can progress to nonalcoholic steatohepatitis and fibrosis. Transient elastography (TE) is a noninvasive liver fibrosis assessment. We evaluated the prevalence of significant liver fibrosis or high liver stiffness measurement (LSM) using the LSM cutoff over 7 kPa and its associated factors in psoriatic patients.* Methods*. Subjects underwent TE and ultrasonography. Univariate and multivariate analysis were performed for the associated factors.* Results*. One hundred and sixty-eight patients were recruited. Three patients were excluded due to TE failure. Mean BMI was 24.8 ± 4.7 kg/m^2^. NAFLD, metabolic syndrome, and diabetes were seen in 105 (63.6%), 83 (50.3%), and 31 (18.8%) patients. The total cumulative dose of methotrexate over 1.5 g was seen in 39 (23.6%) patients. Mean LSM was 5.3 ± 2.9 kPa. High LSM was found in 18 (11.0%) patients. Waist circumference (OR: 1.24; 95% CI: 1.11–1.38; *P* = 0.0002), diabetes (OR: 12.70; 95% CI: 1.84–87.70; *P* = 0.010), and AST (OR: 1.08; 95% CI: 1.02–1.16; *P* = 0.017) were associated with high LSM.* Conclusion*. 11% of psoriatic patients had significant liver fibrosis by high LSM. Waist circumference, diabetes, and AST level were the independent predictors.

## 1. Introduction

Psoriasis is a chronic inflammatory immune-mediated skin disease affecting 1–3% of the world population. It is characterized by increases in local and systemic proinflammatory cytokines [1]. The increased levels of proinflammatory cytokines may explain the progression of atherogenesis and the development of peripheral insulin resistance, which can cause hypertension and diabetes [1, 2]. Recent studies have reported an association between psoriasis and metabolic disorders, such as obesity, dyslipidemia, diabetes, and cardiovascular disorders [1, 3, 4].

Metabolic syndrome is a risk factor for the development of nonalcoholic fatty liver disease (NAFLD). NAFLD is regarded as the hepatic manifestation of insulin resistance and metabolic syndrome. Metabolic syndrome and NAFLD may coexist in psoriatic patients [5]. Lipotoxicity, oxidative stress, cytokines, and proinflammatory mediators may play major roles in the progression from simple steatosis to nonalcoholic steatohepatitis (NASH), advanced fibrosis, and cirrhosis [6]. Psoriasis was reported to be associated with fatty liver and liver fibrosis [7, 8]. Methotrexate is the treatment of choice for moderate-to-severe psoriasis. Unfortunately, liver toxicity is the most important limitation of methotrexate for the treatment of psoriasis [9]. The incidence of methotrexate-related liver toxicity varies considerably depending on concomitant factors such as heavy alcohol drinking. The recently revised consensus stated that psoriatic patients without concomitant diseases should receive liver biopsy when the total cumulative dose of methotrexate reached 3.5–4 g [10]. Theoretically, psoriatic patients with risk factors for NASH may develop liver fibrosis more rapidly and at a lower cumulative methotrexate dose than those without the risk factors. Patients with high risk conditions should undergo liver biopsy when the total cumulative dose of methotrexate exceeds 1.5 g [10]. In addition to methotrexate use, metabolic syndrome and psoriasis-related factors, such as the duration and severity of disease, as well as the presence of joint involvement, may play important roles in the development of significant liver fibrosis [7, 11].

Liver biopsy is the gold standard for assessing the severity of liver fibrosis [12]. However, the risks of biopsy related major complications (0.01–0.1%) limit its use in routine clinical practice [13]. Transient elastography (TE) is a noninvasive tool for the assessment of liver fibrosis that has been evaluated in chronic liver diseases, including psoriasis [14, 15]. To date, the factors that affect the development of significant hepatic fibrosis in psoriasis patients remain unknown, and the prevalence of significant liver fibrosis in Thai patients with psoriasis has never been studied.

The aims of the present study were to investigate the prevalence of significant liver fibrosis or high liver stiffness measurement (LSM) assessed by TE and to evaluate the factors that were associated with high LSM in patients with psoriasis. The objectives of this study were as follows: (i) to study the liver fibrosis in patients with psoriasis by using the TE; (ii) to compare the characteristics of psoriatic patients with and without liver fibrosis.

## 2. Materials and Methods

### 2.1. Patients

After informed consent was obtained, psoriatic patients were enrolled consecutively from the psoriasis clinic at Ramathibodi Hospital, which is a tertiary care medical center, between January 1st and December 31st 2013. Inclusion criteria included an age of ≥18 years and a clinical diagnosis of chronic plaque psoriasis. Exclusion criteria included the presence of known liver diseases such as chronic viral hepatitis, having history of heavy alcohol drinking (>20 g of alcohol per day), or having pregnancy. Data including a detailed history of psoriasis, comorbidities, and regular drug use were collected.

### 2.2. Study Design

A cross-sectional study was conducted in psoriatic patients. The protocol was approved by the hospital ethics committee. The study was carried out according to the Helsinki Declaration (1964) as revised in 2013. Informed consent was obtained from the study participants before enrollment.

### 2.3. Diagnosis of Psoriasis and the Severity Assessment of the Disease

Psoriasis was diagnosed in accordance with the International Classification of Diseases (the 10th revision). Psoriatic arthritis was evaluated based on the criteria recommended by Moll and Wright [16]. The age of onset, duration of disease, and the total cumulative dose of methotrexate were recorded. The psoriasis area and severity index (PASI) score, a score that assesses erythema, induration, and the scaliness of lesions at 4 body areas (the head, trunk, arms, and legs), was calculated [17]. PASI scores range from 0 to 72, where a PASI score of ≥10 was defined as severe psoriasis [17].

### 2.4. Biochemical Testing

Fasting blood tests including alanine aminotransferase (ALT) and aspartate aminotransferase (AST), alkaline phosphatase (ALP), total bilirubin (TB), albumin, gamma-glutamyl transferase (GGT), total, low-density lipoprotein (LDL), and high-density lipoprotein (HDL) cholesterol, total triglycerides (TG), and glucose and insulin levels were collected. Insulin resistance was evaluated based on the homeostatic model assessment insulin resistance (HOMA-IR) index using the formula of fasting plasma insulin (mIU/L) × fasting plasma glucose (mg/dL)/405 [18].

### 2.5. Anthropometric Measurement

Height (m), weight (kg), body mass index (BMI) (kg/m^2^), and waist circumference (cm) were assessed. From the recommendation of the World Health Organization, International Association for the Study of Obesity, and International Obesity Task Force in 2000, the BMI cutoffs of 23.0 to 24.9 and ≥25.0 kg/m^2^ were defined as overweight and obesity in adult Asians [19]. Metabolic syndrome was diagnosed by the consensus definition incorporating International Diabetes Federation (IDF) and American Heart Association/National Heart, Lung, and Blood Institute (AHA/NHLBI) criteria [20]. The consensus definition consists of three of the following criteria: central obesity (waist circumference 102 cm in men and 88 women, or 90 cm in Asian men and 80 cm in Asian women), TG 150 mg/dL or on drug treatment for elevated TG, HDL cholesterol 40 mg/dL in men and 50 mg/dL in women or on drug treatment for reduced HDL, fasting plasma glucose 100 mg/dL or on drug treatment for elevated glucose, and systolic 130 mmHg and/or diastolic 85 mmHg or on antihypertensive drug treatment in a patient with a history of hypertension [20].

### 2.6. Liver Stiffness Measurement Using Transient Elastography (TE)

LSM were assessed by TE (Fibroscan®, Echosen, Paris), which uses an ultrasound transducer that generates vibrations, creating slow elastic shear waves. The median value of LSM was considered to represent the elastic modulus of the liver. TE was performed on the right lobe of the liver through an intercostal space with the patient in the dorsal decubitus position and the right arm maximally abducted. The measured depth of the liver was between 25 and 65 mm. Ten validated measurements were performed. Only the LSM results with 10 validated measurements, a success rate of at least 60%, and an interquartile range (IQR)/median of less than 30% were considered acceptable [21]. The results were expressed in kilopascals (kPa). TE was evaluated by a single well-trained nurse who was blinded to the patient information and had an experience of performing TE in > 1000 cases. The patients underwent TE after at least four hours of fasting [21]. In this study, significant liver fibrosis was defined by high LSM, which used the cutoff of LSM > 7 kPa according to the reports of TE in psoriatic and NAFLD patients [14, 22]. LSM > 9.5 kPa was taken as a cutoff for the presence of advanced liver fibrosis, and LSM > 13.0 kPa was used for the presence of cirrhosis according to previous studies of TE in chronic liver diseases and NAFLD [23, 24]. Patients who had LSM > 7 kPa were encouraged to have liver biopsy but this was not mandatory.

### 2.7. Diagnosis of Fatty Liver by Ultrasonography

The diagnosis and grading of fatty liver were performed with ultrasonography (US) based on the degree of brightness or diffusedly increased echogenicity of the liver parenchyma, echogenic discrepancy of the liver and the kidney, and loss echogenicity of portal venous walls [25].

### 2.8. Statistical Analysis

Sample size calculation was derived from the formula of *N* = *Z*_*α*2_^2^ *PQ*/Δ^2^, given that *α* was 0.05 and *Z*_*α*2_ was 1.96. From a report of significant liver fibrosis in psoriatic patients [14], the prevalence (*P*) was equal to 0.12, and *Q* (or 1 − *P*) was equal to 0.88. As the result, at least 162 psoriatic patients were required to be enrolled in the study. Data were expressed as mean ± SD, median (range), or *n* (%). The means or percentages of baseline data between patients without and with high liver stiffness were compared using Student's *t*-test or Mann-Whitney *U* test for continuous variables and Chi-square test or Fisher's exact test for categorical variables. Factors with a *P* < 0.20 were further selected for multivariate logistic regression analysis. The results are expressed as odds ratios (OR) and 95% confidence intervals (95% CI). A *P* < 0.05 was considered statistical significance. Statistical analysis was performed using SPSS v.22.0 (SPSS Inc., Chicago, USA).

## 3. Results

### 3.1. Demographic and Disease Factors

One hundred and sixty-eight patients were enrolled. Three patients were excluded due to TE failure. The mean BMI was 24.8 ± 4.7 kg/m^2^ (Table 1). About one-third of the participants (32.1%) were overweight, and 20 (12%) patients were obese. The mean waist circumference was 87.0 ± 12.3 cm. Eighty-eight (53.3%), 55 (33.3%), and 31 (18.8%) patients had dyslipidemia, hypertension, and diabetes, respectively. Eighty-three (50.3%) patients met the criteria of metabolic syndrome [20]. Six (3.2%) patients had PASI scores > 10, which was correlated with disease factors reflecting the severity of psoriasis, for example, hospital admission and the requirement of systemic therapy. Only 39 (23.6%) patients had taken methotrexate with the total cumulative dose over 1.5 g. The mean LSM was 5.3 ± 2.9 kPa. Eighteen (10.9%) patients had significant liver fibrosis defined by high LSM or an LSM > 7 kPa. Eleven (6.7%) patients had LSM > 9.5 kPa and 4 (2.4%) patients had LSM > 13.0 kPa, suggestive of advance liver fibrosis and cirrhosis, respectively. Two patients with LSM of 8.9 and 11 kPa underwent liver biopsy. The pathological reports revealed that Metavir fibrotic staging of F2 and F3 without hepatitis was confirmed.

### 3.2. Characteristics of Patients without and with High LSM

The characteristics and laboratory results of the psoriatic patients categorized according to the presence of high LSM are shown in Tables 1 and 2. Univariate analysis was performed to explore factors that were associated with high LSM and the results are shown in Tables 1 and 2. Psoriatic patients without high LSM or significant liver fibrosis (*n* = 147) and those with high LSM or significant liver fibrosis (*n* = 18) had similar characteristics in terms of age and sex (Table 1). Almost all of the psoriatic disease-related factors (the duration and the severity of psoriasis, the presence of psoriatic arthritis, and the total cumulative dose of methotrexate) except for the PASI score were not different between both groups. From univariate analysis, the factors that were significantly elevated in the high LSM group were BMI, waist circumference, the presence of diabetes, hypertension, dyslipidemia, and metabolic syndrome (Table 1). The PASI score was marginally elevated in the high LSM group (*P* = 0.042). The levels of AST, ALT, fasting glucose, and HOMA-IR and the diagnosis of fatty liver were significantly increased in the high LSM group (Table 2).

### 3.3. Factors Independently Related to High LSM

Waist circumference, diabetes, AST level, hypertension, dyslipidemia, PASI score, HOMA-IR index, and the diagnosis of fatty liver by US were selected for further analysis with logistic regression model while avoiding the problem of multicollinearity. The multivariate analysis revealed that waist circumference (OR: 1.24; 95% CI: 1.11–1.38; *P* = 0.0002), diabetes (OR: 12.70; 95% CI: 1.84–87.70; *P* = 0.010), and AST level (OR: 1.08; 95% CI: 1.02–1.16; *P* = 0.017) were independently associated with the presence of high LSM or significant liver fibrosis (Table 3).

## 4. Discussion

Previous studies which were carried out in western countries have revealed that central obesity and metabolic syndrome were highly prevalent in psoriatic patients [2, 3, 5, 7, 8, 26]. Psoriatic patients with central obesity and metabolic syndrome were also at a higher risk of developing liver fibrosis [16, 21, 27].

For psoriasis patients on long-term methotrexate (MTX), serial liver biopsy helps discontinuation of MTX at the time that the significant liver fibrosis appears [28]. The risk of fibrosis progression in MTX associated hepatotoxicity is very low [28]. The necessity of serial liver biopsy should be reconsidered. It may be more reasonable and practical to do serial measures of the amino-terminal propeptide of type III procollagen (PIIINP) every 3–6 months and perform liver biopsy only when PIIINP increases. So, liver biopsy can be avoided in substantial number of psoriatic patients [29, 30].

The study of TE in psoriasis revealed 100% sensitivity for significant fibrosis but only 67% specificity [31, 32]. The combining use of TE and Fibrotest is more accurate for monitoring liver fibrosis in psoriatic patients on MTX [15]. Liver biopsy can be done when abnormal results of both tests present [15]. From a survey in dermatologists worldwide, PIIINP and TE were used by only a minority of dermatologists [33]. TE and Fibrotest should be used in routine clinical practice and both methods should be recommended by medical societies.

The prevalence of significant liver fibrosis or high LSM as defined by an LSM > 7 kPa in Thai psoriatic patients was slightly lower than previous studies (10.9% versus 12–27%) [11, 14]. Similarly, the number of advanced liver fibrosis in psoriasis in this study was slightly lower than a recent report (6.7% versus 8.1%) [24]. Waist circumference (OR: 1.24; 95% CI: 1.11–1.38; *P* = 0.0002) and diabetes (OR: 12.70; 95% CI: 1.84–87.70; *P* = 0.010) were strongly associated with the presence of high liver stiffness or significant liver fibrosis. From this study, we want to highlight that psoriatic patients who had high waist circumference and/or diabetes had the greater risk of developing significant liver fibrosis from TE. Psoriatic patients with diabetes were almost 13 times as likely to have significant liver fibrosis irrespective of other concomitant factors. The influence of diabetes on the progression of liver fibrosis in psoriasis may be explained with the same pathway of insulin resistance and relating cytokines in the pathogenesis of NAFLD and NASH [6]. Noninvasive assessment of liver fibrosis or liver biopsy in psoriatic patients with diabetes should be performed early in their clinical course. The finding of high prevalence of metabolic risk factors (diabetes or central obesity) in psoriatic patients with significant liver fibrosis is consistent with previous reports [15, 29, 34].

The total cumulative dose of methotrexate and old age were not found to be the risk factors of significant liver fibrosis in this study, which is contrasting with the results of previous studies [24, 27]. From some studies, the total cumulative dose of methotrexate was reported to be an important factor for the presence of liver fibrosis [10, 11, 29, 34]. However, there were only 39 (23.6%) patients with the total cumulative dose of methotrexate over 1.5 g in this study. It was suggested that methotrexate might accelerate the progression of NASH [11]. This notion leads to a presumption that psoriatic patients who are on methotrexate may be at a greater risk for the progression of liver fibrosis especially those who have concomitant diabetes or metabolic syndrome or are overweight [11, 35]. Liver fibrosis in psoriatic patients with NASH may develop and progress at a lower cumulative dose of methotrexate than those without NASH.

There were some limitations of our study. Firstly, it was a small sample size and single center study. Larger studies are required to confirm the results of our findings. Secondly, TE which is a noninvasive fibrosis assessment tool was used to measure the degree of liver fibrosis in this study. Liver biopsy, a gold standard tool for liver fibrosis assessment, was performed in only 2 of 18 psoriatic patients who had LSM > 7 kPa.

## 5. Conclusion

Approximately 11% of the psoriatic patients had high liver stiffness or significant liver fibrosis. Our study showed that the presence of significant liver fibrosis in patients with psoriasis was strongly associated with waist circumference, diabetes, and AST level. Other psoriasis-related disease factors and the total cumulative dose of methotrexate could not be demonstrated to be related to the development of significant liver fibrosis in psoriatic patients.

## Figures and Tables

**Table 1:** Demographic data based on the presence of high liver stiffness measurement (LSM > 7 kPa).

	All patients	LSM ≤ 7 kPa	LSM > 7 kPa	*P*
	(*n* = 165)	(*n* = 147)	(*n* = 18)	
Age (years)^†^	49.2 ± 14.0	48.7 ± 14.1	53.4 ± 12.7	0.182
Male^ ‡^	75 (45.5)	65 (44.2)	10 (55.6)	0.454
Body mass index (kg/m^2^)^†^	24.8 ± 4.7	24.0 ± 4.0	30.77 ± 5.7	<0.0001
Waist circumference (cm)^†^	87.0 ± 12.3	85.0 ± 10.7	104.1 ± 12.0	<0.0001
Diabetes mellitus^ ‡^	31 (18.8)	22 (15)	9 (50)	0.001
Hypertension^ ‡^	55 (33.3)	43 (29.3)	12 (66.7)	0.003
Dyslipidemia^ ‡^	88 (53.3)	74 (50.3)	14 (77.8)	0.043
Metabolic syndrome^ ‡^	83 (50.3)	66 (44.9)	17 (94.4)	<0.0001
*Psoriasis disease-related factors*				
Duration of disease (years)^†^	16.5 ± 12.1	16.7 ± 12.3	14.8 ± 10.9	0.533
PASI score^†^	3.0 ± 2.7	2.8 ± 2.6	4.2 ± 3.2	0.042
Severe psoriasis^‡^	6 (3.6)	4 (2.7)	2 (11.1)	0.130
Psoriatic arthritis^ ‡^	35 (21.2)	29 (19.7)	6 (33.3)	0.220
Cumulative dose of MTX^‡^				
None	57 (34.5)	51 (34.7)	6 (33.3)	0.906
≤1.5 g	69 (41.8)	62 (42.2)	7 (38.9)	
>1.5 g	39 (23.6)	34 (23.1)	5 (27.8)	

LSM: liver stiffness measurement; PASI: psoriasis area and severity index; and MTX: methotrexate; ^†^mean ± SD; ^‡^ *n* (%).

**Table 2:** Laboratory results according to the presence of high liver stiffness measurement (LSM > 7 kPa).

	All patients	LSM ≤ 7 kPa	LSM > 7 kPa	*P*
	(*n* = 165)	(*n* = 147)	(*n* = 18)	
AST (U/L)^†^	28.2 ± 13.6	27.1 ± 10.6	37.6 ± 26.7	0.002
ALT (U/L)^†^	41.2 ± 23.0	39.3 ± 19.6	56.4 ± 39.3	0.003
Fasting glucose (mg/dL)^†^	108.4 ± 38.3	103.8 ± 29.7	146.0 ± 69.8	<0.0001
Triglyceride (mg/dL)^†^	126.1 ± 67.8	125.5 ± 68.7	131.3 ± 61.5	0.712
TC (mg/dL)^†^	202.8 ± 42.9	203.6 ± 42.0	194.0 ± 49.9	0.431
LDL-C (mg/dL)^†^	126.1 ± 35.3	126.4 ± 34.3	123.4 ± 44.0	0.781
HDL-C (mg/dL)^†^	50.5 ± 13.2	51.1 ± 13.1	45.4 ± 13.0	0.095
HOMA-IR^†^	3.8 ± 6.1	3.1 ± 3.4	9.9 ± 14.8	<0.0001
LSM (kPa)^†^	5.3 ± 2.9	4.5 ± 1.2	11.5 ± 4.7	<0.0001
Fatty liver by US^‡^	105 (63.6%)	89 (60.5%)	16 (88.9%)	0.019

LSM: liver stiffness measurement; AST: aspartate aminotransferase; ALT: alanine aminotransferase; TC: total cholesterol; LDL-C: low-density lipoprotein cholesterol; HDL-C: high-density lipoprotein cholesterol; HOMA-IR: homeostatic model assessment insulin resistance; US: ultrasonography; ^†^mean ± SD; ^‡^ *n* (%).

**Table 3:** Multivariate logistic regression analysis for the factors associated with high liver stiffness measurement (LSM > 7 kPa).

Variable	Coefficient (*β*)	Standard error	*P* value	Odds ratio	95% CI
Waist circumference	0.21	0.06	0.0002	1.24	1.11 to 1.38
Diabetes	2.54	0.99	0.010	12.70	1.84 to 87.70
AST	0.08	0.03	0.017	1.08	1.02 to 1.16
Dyslipidemia	−1.66	1.09	0.128	0.19	0.02 to 1.62
Fatty liver by US	−1.64	1.23	0.185	0.19	0.02 to 2.18
PASI score	0.14	0.12	0.229	1.15	0.92 to 1.44
HOMA-IR	0.08	0.09	0.329	1.09	0.92 to 1.29
Hypertension	0.81	0.84	0.337	2.25	0.43 to 11.71

LSM: liver stiffness measurement; AST: aspartate aminotransferase; PASI: psoriasis area and severity index; HOMA-IR: homeostatic model assessment insulin resistance; US: ultrasonography.

## Abbreviations

NAFLD: Nonalcoholic fatty liver disease
NASH: Nonalcoholic steatohepatitis
TE: Transient elastography
PASI: Psoriasis area and severity index
HOMA-IR: Homeostatic model assessment insulin resistance
BMI: Body mass index
LSM: Liver stiffness measurement
US: Ultrasonography
OR: Odds ratio.
